# Central Administration of C-X-C Chemokine Receptor Type 4 Antagonist Alleviates the Development and Maintenance of Peripheral Neuropathic Pain in Mice

**DOI:** 10.1371/journal.pone.0104860

**Published:** 2014-08-13

**Authors:** Xin Luo, Wai Lydia Tai, Liting Sun, Qiu Qiu, Zhengyuan Xia, Sookja Kim Chung, Chi Wai Cheung

**Affiliations:** 1 Department of Anaesthesiology, The University of Hong Kong, HKSAR, China; 2 Department of Anatomy, The University of Hong Kong, HKSAR, China; 3 Research Center of Heart, Brain, Hormone and Healthy Aging, The University of Hong Kong, HKSAR, China; 4 Laboratory and Clinical Research Institute for Pain, The University of Hong Kong, HKSAR, China; The James Cook University Hospital, United Kingdom

## Abstract

**Aim:**

To explore the roles of C-X-C chemokine receptor type 4 (CXCR4) in spinal processing of neuropathic pain at the central nervous system (CNS).

**Methods:**

Peripheral neuropathic pain (PNP) induced by partial sciatic nerve ligation (pSNL) model was assessed in mice. Effects of a single intrathecal (central) administration of AMD3100 (intrathecal AMD3100), a CXCR4 antagonist, on pain behavior and pain-related spinal pathways and molecules in the L3-L5 spinal cord segment was studied compare to saline treatment.

**Results:**

Rotarod test showed that intrathecal AMD3100 did not impair mice motor function. In pSNL-induced mice, intrathecal AMD3100 delayed the development of mechanical allodynia and reversed the established mechanical allodynia in a dose-dependent way. Moreover, intrathecal AMD3100 downregulated the activation of JNK1 and p38 pathways and the protein expression of p65 as assessed by western blotting. Real-time PCR test also demonstrated that substance P mRNA was decreased, while adrenomedullin and intercellular adhesion molecule mRNA was increased following AMD3100 treatment.

**Conclusion:**

Our results suggest that central (spinal) CXCR4 is involved in the development and maintenance of PNP and the regulation of multiple spinal molecular events under pain condition, implicating that CXCR4 would potentially be a therapeutic target for chronic neuropathic pain.

## Introduction

Chemokine receptors have been intensively studied for their roles in nociception and considered novel targets for neuropathic pain therapy [1]. C-X-C chemokine receptor type 4 (CXCR4) is the receptor of chemokine (C-X-C motif) ligand 12 (CXCL12), which belongs to the G protein-coupled receptor (GPCR) family. CXCR4 is widely expressed in the peripheral and central nervous system (PNS and CNS) and exerts numerous important functions, such as modulation of neurotransmission, synaptic plasticity, and neuroglial interactions [2]. Increasing number of studies reported roles of CXCR4 in pain processing in the PNS, as CXCR4 is expressed on primary sensory neurons, satellite cells, Schwann cells, and endothelial cells in the peripheral nociceptive structure [3]–[9]. Recent immunohistochemistry studies also showed that CXCR4 would be involved in the modulation of pain signaling in the CNS. CXCL12 and CXCR4 were positive in neurons, astrocytes, microglia/macrophages, and leukocytes in the lumbar spinal cord and their spinal immunoreactivity was found to be increased in a central neuropathic pain model [10]. The activation of spinal CXCR4 by the intrathecal administration of CXCL12 has been found to induce mechanical allodynia for 3 days, which could be reversed by a CXCR4-neutralizing antibody given intrathecally [9].

Currently, systematic (intraperitoneal) administration of CXCR4 antagonist, AMD3100, was demonstrated to have analgesic effects on opioid-induced hyperalgesia [8] and neuropathic pain induced by peripheral neuropathy [3] and by anti-AIDS therapy [6], [7]. These studies implicated the potential application of AMD3100 for analgesia. However, central roles of CXCR4 in pain transduction remain unclear and there has been no study evaluating the effects of pharmacological inhibition of CXCR4 on central pain signal processing. Therefore, in this study, we aimed to explore the roles and mechanisms of central CXCR4 in pain modulation using specific CXCR4 antagonist AMD3100 and a peripheral neuropathic pain (PNP) model using partial sciatic nerve ligation (pSNL) in mice.

## Materials and Methods

### Animals

This research was approved by the Committee on the Use of Live Animals in Teaching and Research (CULATR) (Permit Number: 2610-11), the University of Hong Kong, and performed according to the guidelines for the care and use of laboratory animals as established by the Laboratory Animal Unit (LAU) at the University of Hong Kong. The mice were housed at 23±3°C, with humidity ranges between 25% and 45% under a 12-hour light/12-hour dark cycle (lights on at 07:00). The mice were offered free access to water and food. They were fed with Lab Diet 5012 (1.0% calcium, 0.5% phosphorus, and 3.3 IU/g of vitamin D3).

The experiments were conducted using adult male C57BL/6 wild-type mice (25–30 grams). Total number of mice used in this project was 86. In Rotarod test, 20 mice that had not received any operation or behavior test were randomly divided into four groups (*n* = 5 in each group). The mice of four groups were treated intrathecally with AMD3100 1 µg (AMD3100 1 µg group), 5 µg (AMD3100 5 µg group), 25 µg (AMD3100 25 µg group) or saline (saline group). To study the roles of CXCR4 in the development of PNP, the mice were randomly divided into four groups. Two groups received either pSNL (pSNL group, *n* = 6) or sham surgery (sham group, *n* = 6) without intrathecal drug administration. The other two groups were treated 1 hour before pSNL surgery with a single intrathecal injection of either AMD3100 (5 µg, AMD3100 + pSNL group, *n* = 12) or saline (saline + pSNL group, *n* = 12). To study the roles of CXCR4 in the maintenance of PNP, mice were randomly divided into four groups, and all received pSNL surgery. On post-operative day (POD) 7, four groups were treated with a single intrathecal injection of AMD3100 or of saline. The dosages given to the four groups were 1 µg (pSNL + AMD3100 1 µg group, *n* = 5), 5 µg (pSNL + AMD3100 5 µg group, *n* = 6), 25 µg (pSNL + AMD3100 25 µg group, *n* = 6), or saline (pSNL + saline group, *n* = 5). To study the effects of a single intrathecal injection of AMD3100 (intrathecal AMD3100) on pain pathways in the lumbar spinal cords of pSNL-injured mice, the mice were treated with a single injection of either AMD3100 25 µg (AMD3100 group) or saline (saline group) on POD 7 (*n* = 8 per group). To study the effects of intrathecal AMD3100 on pain molecules in the lumbar spinal cords of pSNL-injured mice, the mice were treated with a single injection of either AMD3100 25 µg (AMD3100 group) or saline (saline group) on POD 7 (*n* = 6 per group).

### Partial sciatic nerve ligation surgery

A pSNL surgery was used as the PNP model in this study [11]. The mice were anesthetized with halothane in a 1∶1 mixture of O_2_ and N_2_O. Under aseptic conditions, the right sciatic nerve (SN) was exposed by an incision from the right sciatic notch to the distal thigh. The location of the SN ligation was identified using the femoral head as a landmark. Approximately 1/2 of the SN was tightly ligated with a 7-0 silk suture. In the sham-operated mice that were used as controls, the right sciatic nerve was exposed, but not ligated. The incision was closed with 5–0 cotton suture and disinfected with ethanol.

### Drug administration

AMD3100 (Sigma), a CXCR4 antagonist, was prepared in saline on the day of the experiment. As the dosage of AMD3100 for intrathecal administration in mice has not been determined, dosages of 1 µg, 5 µg, and 25 µg were used in this study. AMD3100 was administrated as a single dose intrathecally, according to a previous study [12]. Before the injection, the animals were anesthetized using halothane with mixture of oxygen and N_2_O as described above. Using a syringe (Hamilton) with a 30-gauge needle, a spinal cord puncture was made between the L3 and L5 levels in order to deliver a total volume of 5 µl to the subarachnoid space. Successful administration was identified by tail swinging or a tail configuration of the “S” type immediately after injection.

### von Frey test

Mechanical allodynia is the symptom of PNP, which is indicated by a reduction in the paw withdrawal threshold (PWT). This condition was assessed by von Frey test which was performed in our previous research [13]. For habituation before the experiment, the mice were placed in a transparent plastic dome with a metal mesh floor for 20 to 30 minutes. Their PWT was measured with a series of von Frey filaments (IITC). The filaments were applied perpendicularly to the plantar surface of both hind paws, with sufficient force to bend the filaments into an “S” shape. Quick withdrawal or licking of the paw was considered a positive response.

### Rotarod test

To assess whether there would be any the potential side-effect of a single intrathecal injection of AMD3100 (intrathecal AMD3100) on motor function, a rotarod test was conducted to measure the motor coordination and balance of the mice._ENREF_18 Twenty mice that had not received any operation or behavior test were randomly divided into four groups. These animals were habituated to the rotarod apparatus (IITC) for 2 consecutive days at low-speed rotation (5 rpm) for 600 seconds each day before basal measurement. During the experiment, the animals were tested in three accelerating trials of 300 seconds, with the rotarod speed increasing from 5 to 40 rpm over 300 seconds, and with an inter-trial interval of at least 20 minutes. The animals' latency in falling from the rod was recorded for each trial, with a cutoff time at 300 seconds.

### Western blotting

The sciatic nerve of C57BL/6 mice is composed from elements of L3 and L4 segments of spinal cord with a smaller contribution from L5 segment [14]. Therefore, after euthanizing each mouse with pentobarbital, the L3-L5 section of its spinal cord was quickly removed and homogenized in ice-cold RIPA lysis buffer. After concentration determination, protein samples were separated in 10% SDS-PAGE and electrotransferred onto PVDF membranes. Protein samples were then probed with antibodies for extracellular signal-regulated kinase (ERK, 4695, Cell Signaling), phosphorylated ERK (pERK, 4511, Cell Signaling), c-Jun N-terminal kinase (JNK, 9258, Cell Signaling), phosphorylated JNK (pJNK, 4668, Cell Signaling), p38 (9212, Cell Signaling), phosphorylated p38 (p-p38, 4511, Cell Signaling), AKT (9272, Cell Signaling), phosphorylated AKT (pAKT, 4060, Cell Signaling), Signal transducer and activator of transcription 3 (STAT3, 9132, Cell Signaling), phosphorylated STAT3 (pSTAT3, 9131, Cell Signaling), or p65 (4764, Cell Signaling) with alpha-tubulin (T5168, Sigma) used as a loading control. The membranes were visualized with horseradish peroxidase (HRP)-conjugated secondary antibodies (anti-rabbit IgG and anti-mouse IgG, Sigma).

### Real time PCR

After euthanizing each mouse with pentobarbital, the L3-L5 section of its spinal cord was quickly removed and stored at −80°C until RNA extraction. mRNA was extracted from homogenized spinal cord segements following the manufacturer's protocol (Takara). After the quality and quantity determination, mRNA samples were made to cDNA library with reverse transcriptase (Life Technologies). Prepared cDNA samples were diluted and stored at −20°C until further tests. Real time PCR reactions were performed with the SYBR green chemistry method or the Taqman chemistry method (Applied biosystems). The specific primers for substance P (SP), adrenomedullin (AM), calcitonin gene-related peptide (CGRP) and 18S rRNA were reported in our previous study [13], and used with Fast SYBR green master mix (Applied Biosystems). The Taqman Probes for mouse Tumor necrosis factor-α (TNF-α, Mm00443258), interleukin-1β (IL-1β, Mm00434228), interleukin-6 (IL-6, Mm00446190), intercellular adhesion molecule (ICAM, Mm00516023), vascular cell adhesion molecule (VCAM, Mm00449197), excitatory amino-acid transporter-2 (EAAT-2, Mm00441457) and actin-β (Mm01205647) were purchased from Applied Biosystems.

### Statistical analysis

The data were expressed as means ± SEM. Student's *t* tests was used to analyze Western blot and real time PCR data. Results from von Frey test was analyzed using a student's *t* test or a two-way analysis of variance (ANOVA), followed by a Tukey's multiple comparison post test. Results from the rotarod test were analyzed using a two-way analysis of variance (ANOVA), followed by a Tukey's multiple comparison post test. In all cases, *p*<0.05 was considered as statistically significant.

## Results

### Effects of intrathecal AMD3100 on the motor function of mice

**Figure 1:**
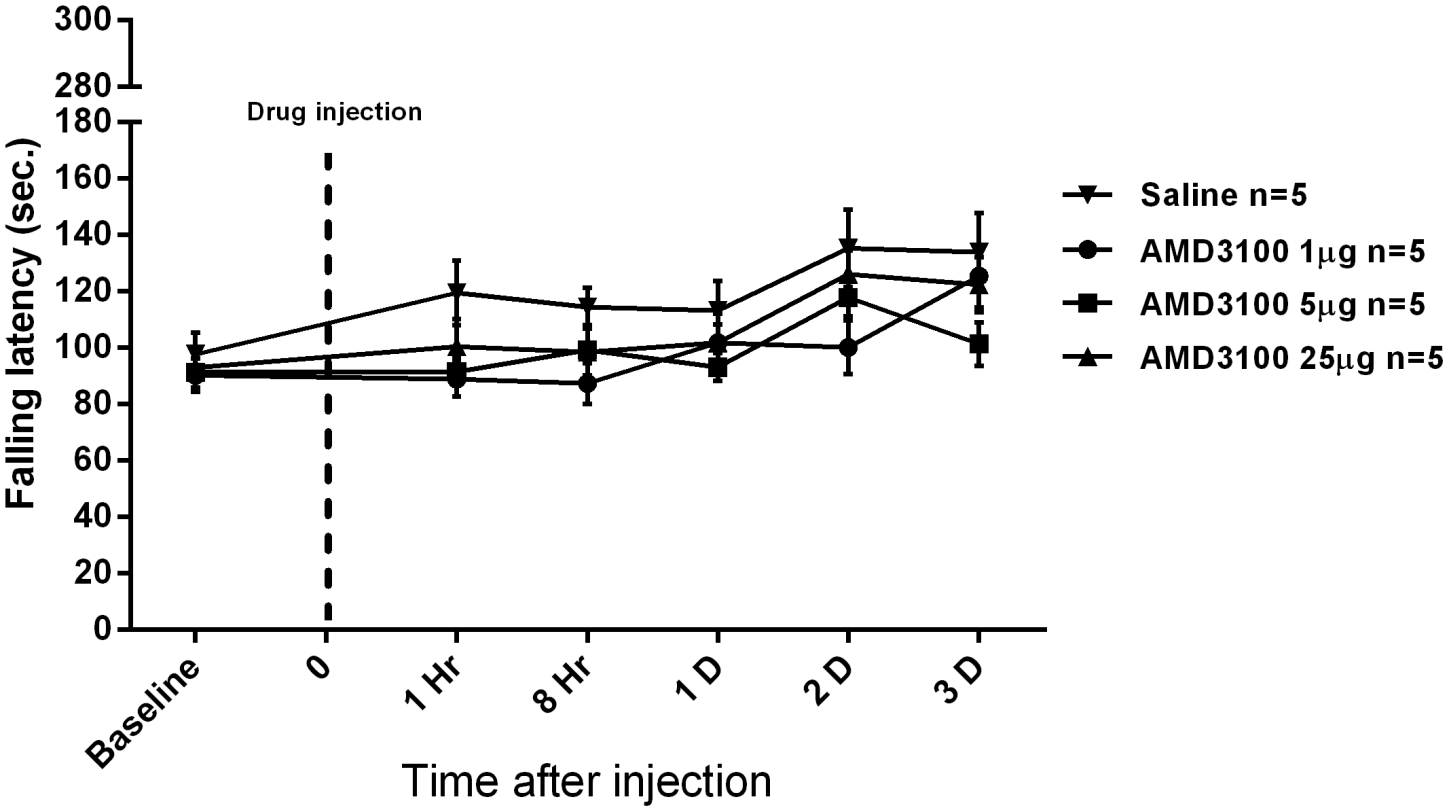
The effects of intrathecal AMD3100 on the motor function of normal mice. There was no significant change in falling latency among the three groups with intrathecal AMD3100, compared to the saline group from hour 1 to day 3 after the injection. Results are mean ± SEM (*n* = 5). Sec.  =  second, D  =  day, Hr  =  hour, 0  =  time of intrathecal injection.

The effects of intrathecal AMD3100 on motor function had not been studied before. Any impairment in motor function might affect the assessment of pain and intrathecal AMD3100 would not be suitable for potential analgesic treatment if that was the case. Therefore, motor function after intrathecal AMD3100 was assessed by a rotarod test in this study. The falling latency of the three groups with intrathecal AMD3100 showed no significant change compared to the saline group after the injection (*p*>0.05; ANOVA; n = 5; Figure 1). These results indicated that intrathecal AMD3100 did not influence the motor function of mice.

### Effects of intrathecal AMD3100 on the development and maintenance of mechanical allodynia in pSNL-injured mice

**Figure 2:**
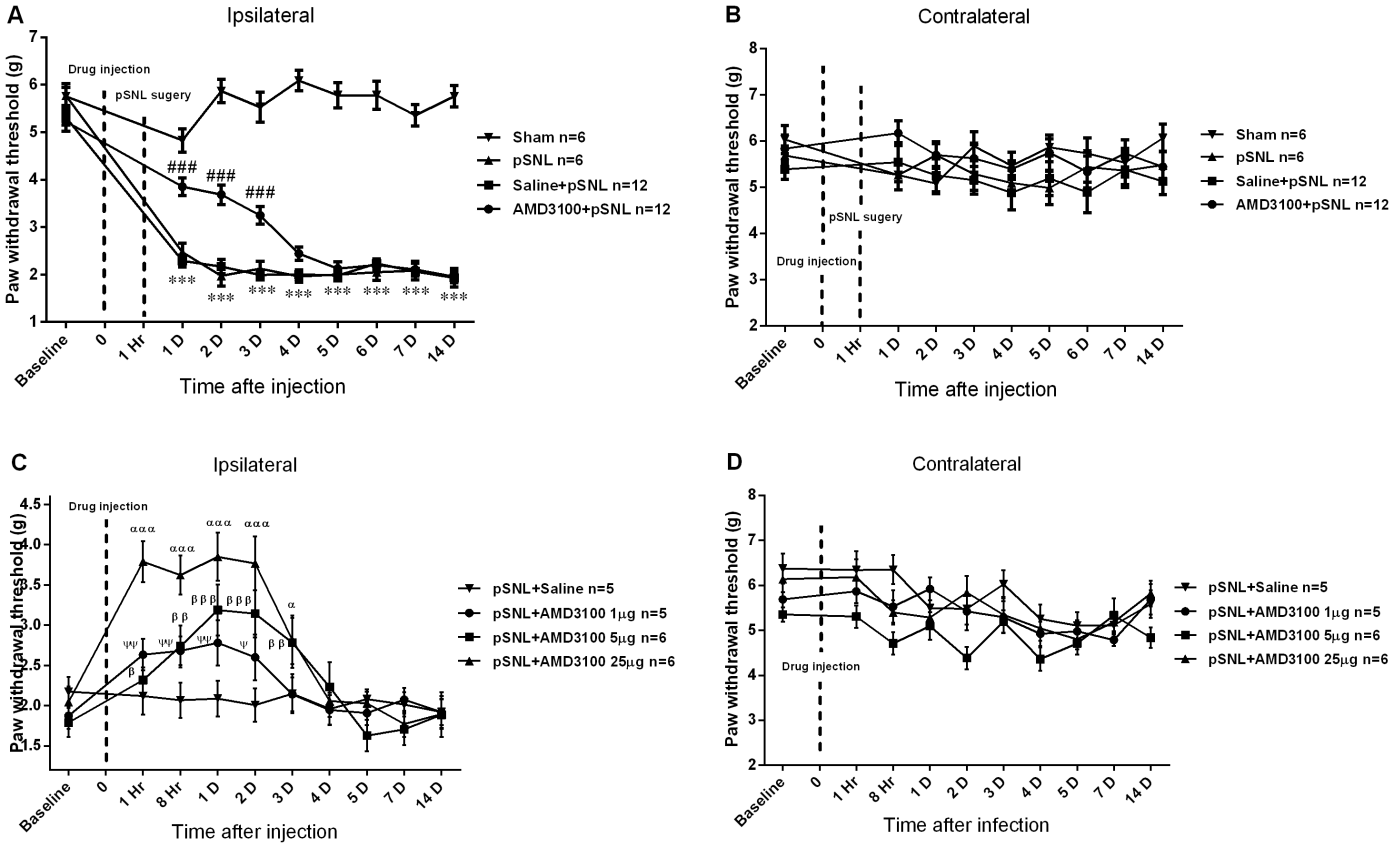
The effects of intrathecal AMD3100 on the development and maintenance of PNP in pSNL-injured mice. The preemptive intrathecal AMD3100, but not intrathecal saline, increased ipsilateral PWT of pSNL mice for 3 days after the surgery (A), but did not affect contralateral PWT (B). On POD 7, intrathecal AMD3100 at dosages of 1 µg, 5 µg and 25 µg increased the ipsilateral PWT of pSNL mice for 2, 3 and 3 days, respectively (C), but did not affect contralateral PWT (D). Results are mean ± SEM (*n* = 5–12). ***p<0.001 versus the pSNL group at the corresponding timepoints. ^###^ *p*<0.001 versus the Saline + pSNL group at the corresponding timepoints. ^ααα^ *p*<0.001, ^α^ *p*<0.05, ^βββ^ *p*<0.001, ^ββ^ *p*<0.01, ^β^ *p*<0.05, ^ΨΨ^ *p*<0.01 and ^Ψ^ *p*<0.05 versus the baseline. g  =  gram, D  =  day, Hr  =  hour, 0  =  time of intrathecal injection.

AMD3100 or saline was given intrathecally at one hour before pSNL surgery, and the involvement of central CXCR4 in the development of PNP was evaluated by von Frey tests. Sham surgery (n = 6) did not affect ipsilateral PWT, but pSNL surgery (n = 6) decreased ipsilateral PWT significantly on POD 1 (*p*<0.001; ANOVA). These results demonstrated that mechanical allodynia was induced immediately by pSNL, but not by sham operation. Pretreatment of a single intrathecal injection of saline (intrathecal saline) did not have any effect on ipsilateral PWT in the pSNL + saline group (n = 12), as compared with that in the pSNL group without any intrathecal medication (*p*>0.05; ANOVA). However, intrathecal AMD3100 before pSNL surgery increased ipsilateral PWT significantly from day 1 to day 3 (*p*<0.001; ANOVA; n = 12) after drug injection, as compared with the pSNL + saline group (Figure 2A). No difference in contralateral PWT was found among the four study groups (*p*>0.05; ANOVA; Figure 2B). These results suggested that intrathecal AMD3100 could delay the development of mechanical allodynia by pSNL.

To study the role of central CXCR4 in the maintenance of PNP, pSNL-injured mice received either intrathecal AMD3100 or intrathecal saline on POD 7. Mechanical allodynia from hour 1 to day 14 after the injection was assessed by von Frey test. Intrathecal AMD3100 1 µg increased ipsilateral PWT from hour 1 to day 2 (*p*<0.01 or 0.05; student's *t* test; n = 5; Figure 2C) after the injection. Intrathecal AMD3100 5 µg increased ipsilateral PWT from hour 1 to day 3 (*p*<0.001 or 0.01 or 0.05; student's *t* test; n = 6; Figure 2C) after the injection. Intrathecal AMD3100 25 µg increased ipsilateral PWT from hour 1 to day 3 (*p*<0.001 or 0.05; student's *t* test; n = 6; Figure 2C) after the injection. Intrathecal saline did not have any effect on ipsilateral PWT (*p*>0.05; student's *t* test; n = 5; Figure 2C). No significant difference was shown in contralateral PWT among any of the four groups (*p*>0.05; student's *t* test; Figure 2D). These results suggested that intrathecal AMD3100 could transiently reverse established mechanical allodynia by pSNL in a dose-dependent way.

### Effects of intrathecal AMD3100 on the production of pain molecules in the lumbar spinal cord of pSNL-injured mice

**Figure 3:**
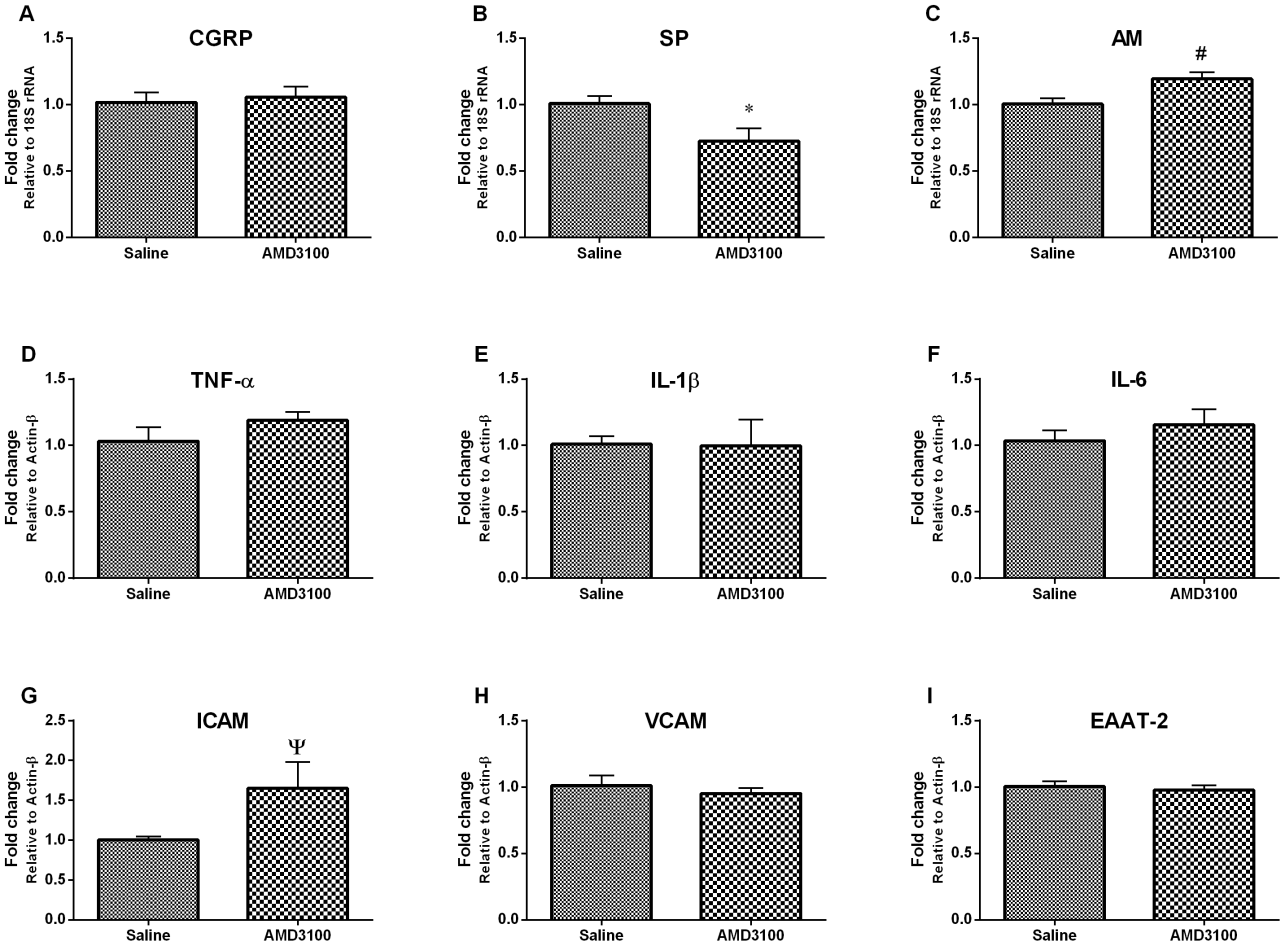
The effects of intrathecal AMD3100 on the production of neuropeptides and pro-inflammatory cytokines, and cellular adhesion molecules in the ipsilateral L3-L5 spinal cord segment of pSNL-injured mice. Real time PCR revealed that intrathecal AMD3100 decreased mRNA level of SP (B) and increased mRNA level of AM (C) and ICAM (G), but did not affect mRNA levels of CGRP (A), TNF-α (D) and IL-1β (E), IL-6 (F), VCAM (H) or EAAT-2 (I) at hour 8 after the injection. Results are means ± SEM (n = 6). *p<0.01, ^#^ *p* and ^Ψ^ *p*<0.05 versus the saline group.

Following the peripheral nerve injury, the central release of neuropeptides (e.g. SP, CGRP and AM), pro-inflammatory cytokines (e.g. TNF-α, IL-1β and IL-6), cellular adhesion molecules (e.g. ICAM and VCAM) and glutamate transporter (e.g. EAAT-2) contribute to neuronal central sensitization and persistent pain [15], [16]. Therefore, we also investigated whether intrathecal AMD3100 could affect the spinal production of these neuropeptides and pro-inflammatory cytokines. The mRNA level of SP was decreased but the mRNA level of AM and ICAM were increased significantly on the ipsilateral side of the L3-L5 spinal cord segment in pSNL-injured mice with intrathecal AMD3100 25 µg (n = 6) compared to the saline group (n = 6) at 8 hour after the injection (*p*<0.05; student's *t* test; Figures 3B, C and G). However, the mRNA levels of CGRP, TNF-α, IL-1β, IL-6, VCAM and EAAT-2 in the L3-L5 spinal cords of pSNL-injured mice were not affected by intrathecal AMD3100 (all *p*>0.05; student's *t* test; Figures 3A, D, E, F, H and I).

### Effects of intrathecal AMD3100 on pain signaling pathways in the lumbar spinal cord of pSNL-injured mice

**Figure 4:**
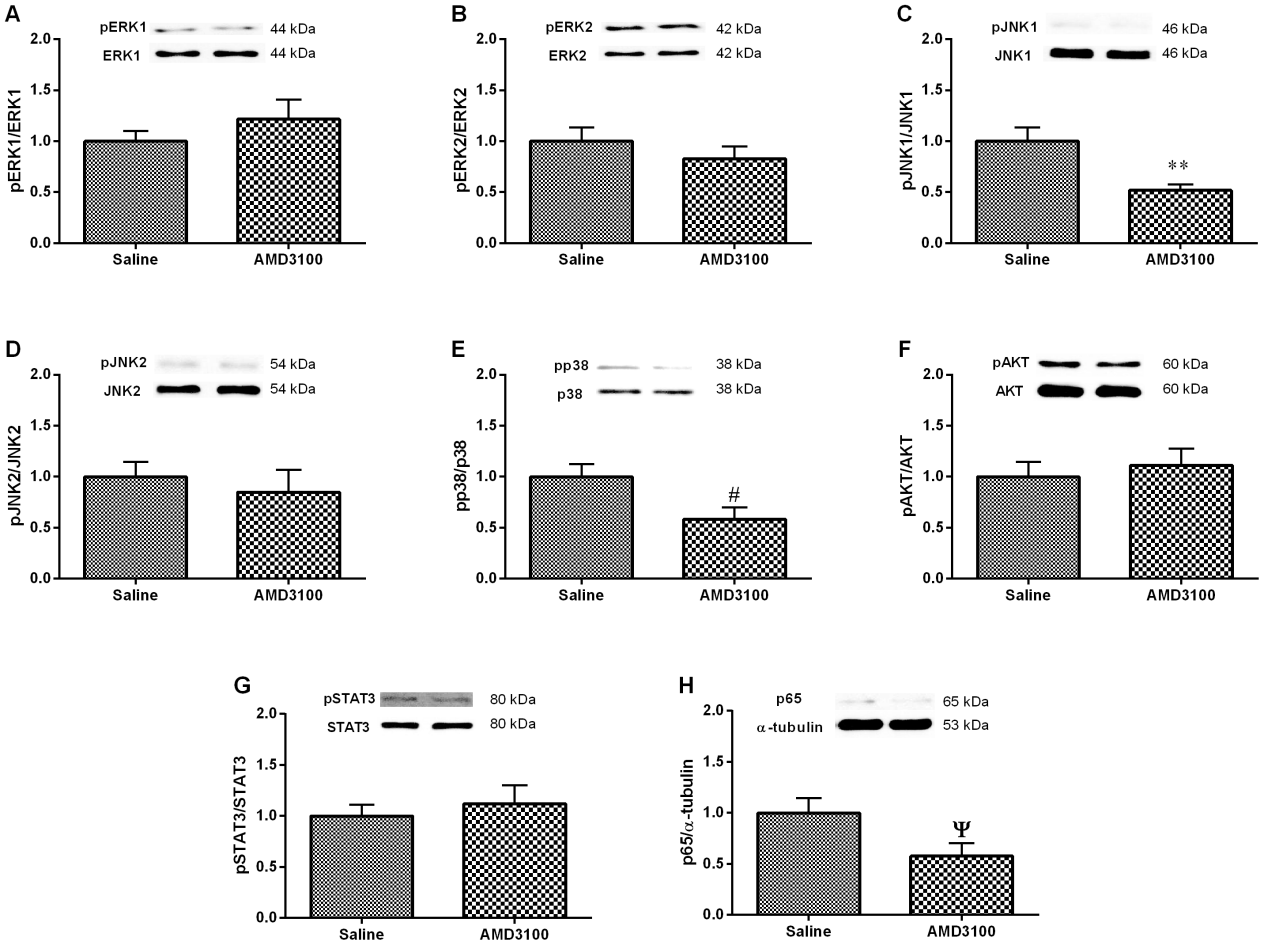
The effects of intrathecal AMD3100 on pain pathways in the ipsilateral L3-L5 spinal cord segment of pSNL-injured mice. Western blotting revealed that intrathecal AMD3100 decreased the phosphorylation level of the JNK1 (C), p38 (E) and p65 (H) pathway, but did not affect the phosphorylation levels of the ERK1 (A), ERK2 (B), JNK2 (D), AKT (F) or STAT3 (G) at hour 8 after the injection. Results are means ± SEM (n = 8). **p<0.01, ^#^ *p* and ^Ψ^ *p*<0.05 versus the saline group.

Spinal mitogen-activated protein kinases (MAPKs), phosphoinositide 3-kinase (PI3K), STAT3 and nuclear factor kappa-light-chain-enhancer of activated B (NF-κB) pathways were shown to be significantly activated on POD 7 using PNP models [17]–[20]. Our result found that intrathecal AMD3100 in all used dosage produced analgesic effects on pSNL-injured mice at hour 8 after the drug injection. Therefore, we harvested lumbar spinal cord sample from this time-point and detected whether intrathecal AMD3100 could affect the activation of members of these pathways. The phosphorylation level of JNK1 (*p*<0.01; student's *t* test; Figure 4C) and p38 (*p*<0.05; student's *t* test; Figure 4E) and the expression level of p65 (*p*<0.05; student's *t* test; Figure 4H) was decreased significantly on the ipsilateral side of the L3-L5 spinal cord segment in pSNL-injured mice with intrathecal AMD3100 25 µg (n = 8), compared to the saline group (n = 8). However, the activation (phosphorylation) levels of ERK1, ERK2, and JNK2 in the L3-L5 spinal cords of pSNL-injured mice were not affected by intrathecal AMD3100 (all *p*>0.05; student's *t* test; Figures 4A, B, D, F and G).

## Discussion

Although recent immunohistochemistry study has suggested the involvement of CXCR4 in pain processing in the CNS [10], central roles of CXCR4 in nociception remain unclear. Therefore, in order to explore the function of CXCR4 in pain modulation in CNS, we studied the pharmacological effects of intrathecal administration of CXCR4 antagonist (AMD3100) on PNP using a pSNL model. The pSNL model is a widely used PNP model, and long-lasted mechanical allodynia is induced following partial sciatic ligation [11]. Our present work employed a single intrathecal injection of AMD3100 before pSNL surgery or on POD 7 with similar methods used in antagonism studies of other chemokine receptors [21], [22]. Evidence for the involvement of central CXCR4 in the initiation of PNP came from the observation that the pretreatment of AMD3100 led to an attenuation of pSNL-induced allodynia. Essentially, this CXCR4 antagonist was also effective in alleviating mechanical allodynia when pSNL-induce neuropathy had been established. The results indicated that CXCR4 also contribute to the early maintenance of PNP state. Moreover, the reliability of these behavioral anti-nociceptive effects was enhanced by the facts the intrathecal AMD3100 did not impair any motor function.

Previous studies reported analgesic effects of systematic administration of AMD3100 only lasted for hours [8], [10], but the effects of central administration of AMD3100 as used in our current study could last for several days. As CXCR4 is widely distributed on nearly all cell types and plays important roles in the tissue development [23], systematic (intraperitoneal) administration of CXCR4 antagonist would be of lower nociceptive structure-specificity and anti-nociceptive efficiency relative to central (intrathecal) administration of AMD3100 theoretically. Moreover, intrathecal delivery of AMD3100 is highly specific to spinal cord, implicating that central (spinal) administration of CXCR4 antagonist for analgesia would be more effective than peripheral injection. While the effects of AMD3100 on DRG have been previously reported [3], [4], [6]–[8], its spinal effects have not been studied. Multiple mechanisms would accounts for the anti-nociceptive effects of intrathecal AMD3100, as CXCR4 is expressed on neuron, glia and other cell types in the lumbar spinal cord [9], [10]. Therefore, in the current study, we explored its effects on well-known pain pathways and molecules at the lumbar spinal cord.

GPCR coupled signaling, including MAPKs, PI3K, STAT3 and NF-κB pathways, are proposed to function in CXCR4 signaling, and these pathways are also activated in the lumbar spinal cord following peripheral neuropathy [17]–[20]. As these pathways are activated by different cell type-specific mechanisms, antagonism study of intrathecal AMD3100 on these pathways can explain how CXCR4 signaling works on PNP in the CNS. The present study found that, among MAPK pathways, intrathecal AMD3100 downregulated spinal phosphorylation level of JNK1 and p38, but did not affect ERK1, ERK2 or JNK2. Peripheral neuropathy activates ERK1/2 in neuron, astrocyte and microglia. pSNL-induced activation of JNK1/2 is restricted to spinal astrocyte, while, the activation of p38 is restricted to spinal microglia [17]. This study also uncovered that intrathecal AMD3100 reduced the spinal expression of p65. The family of NF-κB transcription factors is associated with neuropathic pain, and the activation (increase in expression) of these factors is selectively restricted in spinal glial cells (astrocyte and microglia) [18], [24]. Peripheral neuropathy also activates STAT3 (restricted to spinal microglia [20]) and AKT (restricted to spinal neuron [19]); however our present study did not found any effect of intrathecal AMD3100 on STAT3 or AKT pathways. Therefore, this research found that, among pain-related CGRP pathways, some pathways activated in spinal glial cells would account for anti-nociceptive effects of intrathecal AMD3100.

This research also studied the effects of intrathecal AMD3100 on pain-related molecules. Our study showed that AMD3100 given intrathecally reduced the spinal production of SP and increased that of AM, but its effects on CGRP were not detectable. SP is locally expressed by secondary sensory neuron at the lumbar spinal cord [25], and spinal SP expression is inducible following neuropathy, implicating that SP may account for the pain signaling to higher level of nociceptive structure [26]. AM is a pain-related neuropeptide with partial homology to CGRP, and recent research with transgenic technology unveiled its central role in pain processing was more complicated than previously surmised [27]. Because the presence of pre-synaptic and post-synaptic CXCR4 receptor implicates the potential interaction of CXCR4 signaling and the release of neurotransmitters [9], our results verified this hypothesis that neuronal CXCR4 may modulate SP and AM-mediated synaptic activity in central nociception. TNF-α, IL-1β and IL-6 are mainly released by spinal glial cells and contribute to the alteration of pain processing centrally [28]. Spinal production of these cytokines was transiently increased at early stage following peripheral neuropathy, implicating that these pro-inflammatory cytokines would be important to the development, but not the maintenance, of neuropathic pain [29]. Therefore, our results indicated that effects of intrathecal AMD3100 on maintenance of PNP did not involve the regulation of these pro-inflammatory cytokines. Moreover, our study found that AMD3100 given intrathecally increased the spinal mRNA level of ICAM. The blood-spinal cord barrier (BSCB) offers a specialized microenvironment for the cellular constituents of the spinal cord, and increasing evidence shows that BSCB is involved in nociception following neuropathic injury [30]. The expression level of ICAM correlates with the permeability of BSCB and the infiltration of immune cells to the CNS [31]. Therefore, this result indicated that central CXCR4 would be involved in the regulation of the permeability of BSCB and the immune response to PNP at the spinal cord. Spinal expression of EAAT-2 is decreased with peripheral neuropahty, and the prevention of downregulated EAAT-2 expression can potentially be an analgesic target [32]. Nevertheless, this studied did not show the involvement of central CXCR4 in the regulation of EAAT-2 expression.

In conclusion, this study firstly reports that central administration of CXCR4 antagonist AMD3100 can attenuate the development and the maintenance of PNP after pSNL without any impairment in motor function. Among well-known pain related pathways and molecules, the analgesic effects of intrathecal AMD3100 involve the downregulation of JNK1, p38 and p65 pathways, with the decrease in SP and the increase in AM and ICAM in the lumbar spinal cord of pSNL-injured mice. This study is an essential step toward understanding the contribution of CXCR4-mediated pain processing in CNS, which can lead to chemokine-targeted analgesia in the future.
